# Mothers treatment seeking intention for neonatal danger signs in northwest Ethiopia: A structural equation modeling

**DOI:** 10.1371/journal.pone.0209959

**Published:** 2018-12-31

**Authors:** Tariku Nigatu Bogale, Abebaw Gebeyehu Worku, Alemayehu Worku Yalew, Gashaw Andargie Bikis, Zemene Tigabu Kebede

**Affiliations:** 1 Institute of Public Health, University of Gondar, Gondar, Ethiopia; 2 School of Public Health, Addis Ababa University, Addis Ababa, Ethiopia; 3 Department of Pediatrics and Child Health, University of Gondar, Gondar, Ethiopia; Harvard TH Chan School of Public Health, UNITED STATES

## Abstract

**Background:**

Neonatal mortality contributes to nearly half of under-five mortality in Ethiopia. Treatment seeking for newborn danger signs remains low despite correlations with neonatal mortality. This study tests a theoretical model of factors affecting mothers’ treatment seeking intention for neonatal danger signs in northwest Ethiopia.

**Method:**

A cross sectional study was conducted from March 3–18, 2016 in northwest Ethiopia. A total of 2,158 pregnant women and women who had delivered in the past 6 months were interviewed. Latent variables; knowledge of neonatal danger signs (KDS), household level women empowerment (HLWE) and positive perception toward the behavior of health care providers (PPBHCP) were measured using a Five Point Likert Scale. Socioeconomic status (SES), number of antenatal care attendance, perceived cost of treatment (PCT), average distance to health facilities (ADHF) and treatment seeking intention (TSI) were observed variables in the study. A structural equation modeling was applied to test and estimate the hypothesized model of relationships among latent and observed variables and their direct and indirect effects on TSI.

**Result:**

KDS, PPBHCP, HLWE, and PCT showed direct, positive and significant association with TSI (β = 0.41, p<0.001, β = 0.08, p<0.002, β = 0.18, p<0.001, and β = 0.06, p<0.002, respectively). SES was not directly associated with TSI. However, it indirectly influenced TSI through three pathways; KDS, number of ANC attendance and HLWE (β = 0.05, p<0.05, β = 0.08, p<0.001 and β = 0.13, p<0.001, respectively). Number of antenatal care was not directly associated with TSI. But indirectly, it affected TSI through its direct effect on KDS and PPBHCP (β = 0.05, p<0.05, β = 0.14, p<0.001, respectively). PPBHCP and HLWE also showed indirect association with TSI through their direct effect on KDS (β = 0.37, p<0.001, β = 0.36, p<0.001, respectively). All in all, the model fitted the sample data and explained 31% of the variance in TSI.

**Conclusion:**

PPBHCP, HLWE, PCT and KDS were associated with mothers’ TSI for newborn danger signs.

## Background

Globally, around 44% of under-five deaths is due to neonatal mortality [1–3]. Around two-third of neonatal deaths occur in just 10 countries[3]. The majority of neonatal deaths occur at home in resource limited settings including Ethiopia[4,5]. Compared to other developing countries, Ethiopia has one of the highest neonatal mortality rates (29 deaths per 1000 live births) contributing to nearly half of under-five deaths in the country[6].

Evidences show that there is correlation between treatment seeking at health facilities and neonatal mortality[7–9]. Meaning, death of newborns is preventable when appropriate and timely care is sought[10]. A study also showed that a third of neonatal deaths can be prevented by caring for small and ill newborns [3]. However, care seeking behavior for neonatal illnesses is low in low and middle income countries [11]. Health seeking behavior and utilization is also affected by several factors including; physical, cultural and political factors [12], low income [13], knowledge about the severity of illness [14], cost of treatment, staff friendliness, poor communication with staff and distance to health facility [15]. Treatment seeking also varies between sexes and places of residence. Treatment seeking for male neonate is better than female neonate[16].

UNICEF and WHO define the following symptoms as danger signs in newborns: 1) Not feeding since birth or stopped feeding, 2) Convulsions, 3) Respiratory rate of 60 or more, 4) Severe chest in-drawing, 5) Temperature ≥ 37.5^0^ C, 6) Temperature ≤ 35.5^0^ C, 7) movement only when stimulated, or not even when stimulated, 8.)Yellow soles (sign of jaundice), 9) Reddened or pus draining umbilicus and, 10) Reddened or pus draining eyes. According to WHO, these symptoms are believed to be easily recognizable by community health workers or possibly mothers [17]. Evidences show that caretakers who don’t recognize these dangers signs fail to seek appropriate treatment for the illnesses [18,19].

The Ethiopian Ministry of Health, through its flagship health extension program, prepared an illustrated booklet called Family Health Card (FHC). The FHC contains recommended action points and key health messages on maternal, newborn and child health. It also contains messages on neonatal danger signs to help families recognize neonatal danger signs and seek prompt treatment [20].

Despite the implementation of the FHC and other newborn focused interventions such as Community Based Newborn Care (CBNC), integrated Community Case Management (iCCM), and Integrated Management of Newborn and Childhood Illnesses (IMNCI), Ethiopia still has one of the highest neonatal mortality rates in the world[21]. Modern treatment seeking for neonatal danger signs is also negatively influenced by social, cultural and religious factors in the country[22–24].

Even though there is evidence of a link between treatment seeking and neonatal mortality, there is scarcity of evidence on the factors that determine treatment seeking for neonatal danger signs. In addition, previous studies assume summative relationships among predicator variables and did not investigate their inter-correlations[16,25–27]. This study, however, hypothesized a theoretical model of treatment seeking intention. The model applies a robust methodology called structural equation modeling that simultaneously tests and estimates effects of variables and their inter-relationships in determining mothers’ treatment seeking intention for newborn danger signs.

## Methods and materials

### Study design, setting and source population

A cross sectional community-based study was conducted from March 3–18, 2016 in North Gondar Zone of Ethiopia. North Gondar is in Amhara region located in the northwest part of the country[28]. The zone has 24 woredas (districts). According to the Central Statistical Agency (CSA), in 2014, the zone’s projected population based on the 2007 national population and housing census was 3,441,885 of which 1,741,549 were males[29]. As of 2016, the zone has 9 government hospitals, 126 health centers and 563 health posts. There are also many private clinics most of them located in urban areas.

### Study population and variables

Pregnant women and women who had delivered (stillbirth or livebirth) in the past 6 months were included in the study. These groups of women are targets of different information on newborn care from formal (e.g. the FHC and health facilities) and informal (e.g. community and family members) sources during and after pregnancy. Women who delivered in the past 6 months were included to reduce memory bias.

### Hypothesized theoretical model

**Fig 1:**
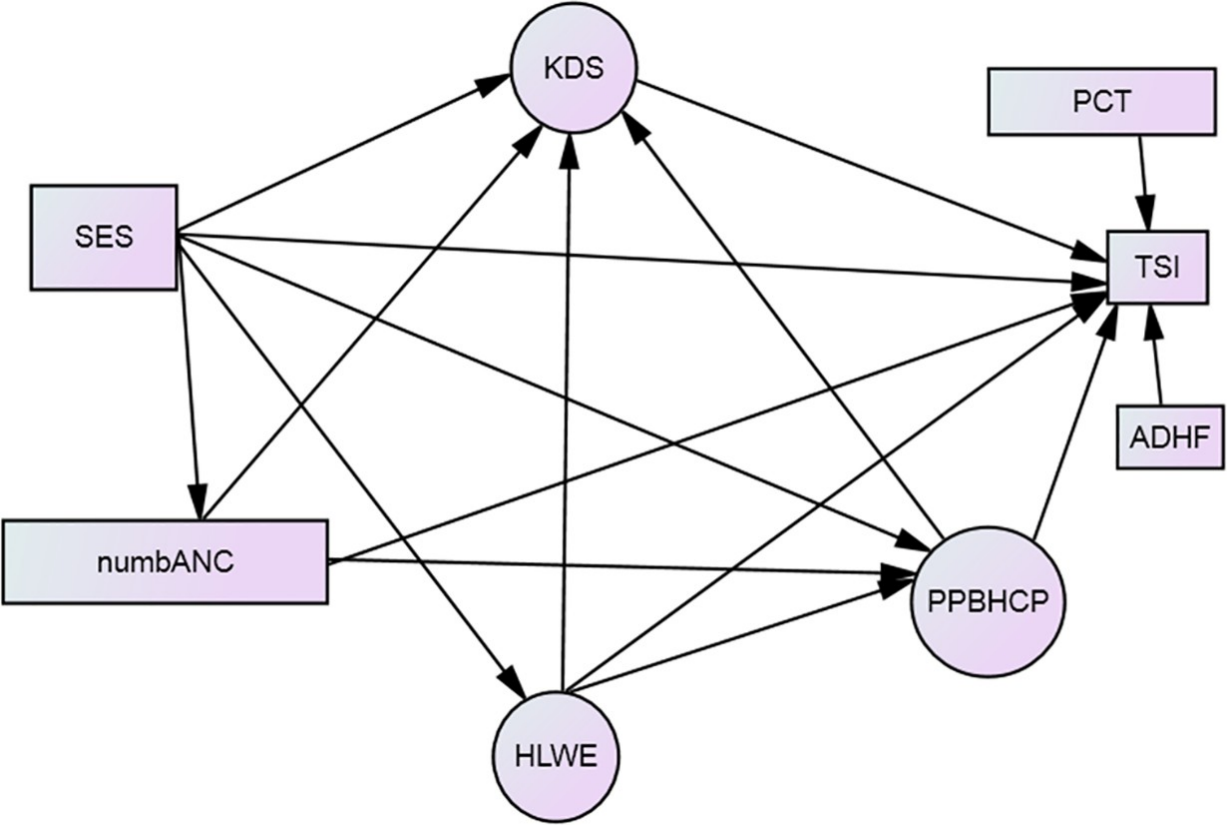
Hypothesized structural model for mother's treatment seeking intention for neonatal danger signs Keys: SES: socioeconomic status, numbANC = number of ANC attendance, KDS = knowledge of danger signs, PCT = perceived cost of treatment, TSI = treatment seeking intention, ADHF = average distance to health facility, PPBHCP = positive perceived behavior of health care providers, HLWE = household level women empowerment.

A hypothesized relationship was constructed among latent and observed variables based on literature review, plausibility of relationship and the authors experience (**Fig 1).** The following evidences supported the development of the hypothesized theoretical model:

Socio-economic status (SES) is associated with caretakers’ treatment seeking [12–14,19,30]. number of ANC visits is associated with knowledge of danger sign [30], wealth status[31], and treatment seeking intention[32] Treatment seeking for newborn illnesses is associated with perceived behavior of health care providers[11,19,33], distance to health facilities [14], cost of treatment [15] and women’s empowerment[34,35].

TSI in this model is used as a proxy measure for treatment seeking behavior[36–38]. The model has three constructs also called latent variables or factors (PPBHCP, HLWE, and KDS) represented by circles and five observed variables (SES, number of ANC attendance, ADHF, PCT and TSI) represented by rectangles. The latent factors represent shared variances of items or indicator variables. Lines indicate relationships between the variables. Lack of a line connecting two variables implies no hypothesized relationship [39].

### Measurements and tools in the questionnaire

#### Measurements

Items for all latent and some observed variables were measured using a Five Point Likert Scale: strongly disagree = 1, disagree = 2, neutral = 3, agree = 4 and strongly agree = 5. The items were asked in affirmative statements to which respondents replied. As scoring increased from 1 to 5, it denoted higher score in the latent and the observed variables. Some items were reverse coded to maintain this assumption.

To help score the Likert Scale easily, respondents were instructed by interviewers to use the five digits of the right hand fingers to select one of the five responses; moving from the little finger to the thumb representing increasing level of agreement; strongly disagree (little finger), disagree (ring finger), neutral (middle finger), agree (index finger), and strongly agree (thumb), respectively. Trained interviewers used their right hands to help each respondent score the Likert Scale using each questions.

#### Latent variables measurement

Knowledge of dangers signs (KDS): Mothers level of agreement with the WHO defined danger signs was measured with ten items. Each item represented one danger sign. The level of agreement to whether a newborn illness symptom defines a danger sign or not was measured using the Five Point Liker Scale. No mention was made to the women whether the symptoms were defined as dangers signs by WHO.

Household level women’s empowerment (HLWE): This was measured by two items; level of partner support and household level women’s decision making (HDM). The items were; (1) My husband/partner is supportive if I make decisions to take my sick newborn to treatment even in his absence, and (2) I can make decisions, alone or with my husband/partner, concerning household resources and my family.

Positive perception toward behavior of health care providers (PPBHCP): PPBHCP was measured by items used to measure respectful and non-abusive facility based care for maternity services[40]. (1) Health providers discriminate patients (reverse coded), (2) Health providers abuse patients (reverse coded), (3) Health providers create unnecessary delays to give care (reverse coded), (4) Health providers give enough time to listen to our worries (5) Health providers explain procedures before treatment and, (6) Health providers give confidential care or services.

#### Observed variables measurement

Socioeconomic status (SES): Principal component analysis (PCA) was used to measure SES. Variables included; ownership of house, household assets (possession of animals, farmland, bank account and utilities), household characteristics (what the floor, the wall and the roof is made of and possession of latrines and its hygiene status),and amenities (presence of electricity, source of drinking water, the light source and the type of fuel the household use). The household characteristics and amenities were dichotomized as improved and not improved and coded as 1 = improved and 0 = unimproved. The SES index was constructed separately for urban and rural areas. The SES indices were weighted for urban and rural areas and then combined to produce a single SES. The SES was ranked in to quintiles and used as observed variable.

Treatment seeking intention (TSI): TSI was measured by the mother’s level of agreement to the item; I always take my newborn to health facility for treatment of illnesses. The illness symptoms that define danger signs were mentioned without telling the respondents that the symptoms were defined as danger signs by WHO. The agreement level was scored using a Five Point Likert Scale.

Perceived cost of treatment (PCT): To measure what cost mothers think newborn danger sign treatment would entail, mothers were asked for their agreement to the item: The cost for the treatment and care for newborn danger signs is fair, or there is no cost. The response was scaled in a range of 1 to 5, similar to TSI.

Average distance to the nearest health facility (ADHF): The average distance to the nearest health post and health center was taken. Distance from health post to each village was obtained from health posts and health centers.

Number of ANC attendance(numbANC): The number of ANC attendance, as reported by mothers, for the current (for pregnant women) or last pregnancy (for those who delivered in the last six months) at a health post or a health center.

### Reliability analysis for measurement items

Reliability analysis was used to check for internal consistency of items used in the measurement of the latent variables. Items with corrected item-to-total correlation of less than 0.30 and alpha if item deleted greater than the overall alpha were used as criteria to delete items. Cronbach’s alpha measures the extent to which all the items in a test measure the same concept or construct and hence it is connected to the inter-relatedness of the items within the test[41].

Higher Cronbach’s alpha value does not guarantee unidimentionality of items[41]. Dimensionality of the items was tested using PCA. All items loaded on a single construct which they were initially supposed to measure. Accordingly, the Cronbachs alpha coefficient for KDS, PBHCP, and HLWE were 0.85, 0.80, and 0.79, respectively.

### Sample size, sampling and data collection techniques

Sample size was determined using estimable parameter to cases ratio of 1:30 to get more stable estimates[42]. There were 57 free estimable parameters in the model. Hence, the sample size was calculated as; 57 multiplied by 30 which equals 1,710. This study was conducted as part of a multicountry enhanced iCCM study which had adequate sample size (n = 2,158).

A multistage cluster sampling technique was used to select pregnant women and women who had delivered (stillbirth or livebirth in the past six months prior to the data collection time. First, 39 kebeles (the smallest administrative units) were randomly selected from three districts (Debark, Dabat and Wogera) proportional to the size of the districts. Then, villages ‘‘Gots” from the selected kebeles were randomly selected. Data was collected from all households “Got” where there were eligible women.

Fifty four data collectors and five supervisors, all of them with at least first degree in health sciences, were recruited and trained for two days. A pretested structured questionnaire was used to collect data.

### Data analysis and modeling

The data was entered into EpiData Version 3.1. The database was exported to IBM SPSS version 20 and cleaning was done by running frequencies and descriptive statistics. The cleaned data was checked for outliers, nonlinearity, and multivariate normality. Analysis of Moment Structure (AMOS), add-in software within SPSS, was used for the analysis.

Internal consistency of the measurement items was assessed using Cronbach’s Alpha. Structural equation modeling (SEM) was used to test and estimate the relationships between variables and their pathways of influence on treatment seeking intention.

The hypothesized model was defined a priori (model specification) based on theory, previous analytic researches and plausibility of relationships (Fig 1). The relationships between variables in the hypothesized model are shown by the following equations:

$$TSI=\beta_{0}+\beta_{1}KDS+\beta_{2}PPBHCP+\beta_{3}SES+\beta_{4}numbANC+\beta_{5}HLWE+\beta_{6}ADHF+\beta_{7}PCT+D_{1}$$

$$KDS=\beta_{00}+\beta_{8}PPBHCP+\beta_{9}SES+\beta_{10}numbANC+\beta_{11}HLWE+D_{2}$$

$$PPBHCP=\beta_{000}+\beta_{12}SES+\beta_{13}numbANC+\beta_{14}HLWE+D_{3}$$

$$HLWE=\beta_{0000}+\beta_{15}SES+D_{4}$$

$$numbANC=\beta_{00000}+\beta_{16}SES+D_{5}$$

The βs (betas) represent the change in the dependent variables for a unit change in the value of the corresponding predictor variable keeping other variables constant. The **Ds** in each equation denote the disturbance; part of the total variance that is not explained by the particular model.

Model identification was checked using the degree of freedom (DF = 219). When DF>0, it means that the model is over identified and unique parameter estimates can be calculated[42]. The regression weights (β coefficients or path coefficients) and factor loadings were determined using maximum likelihood estimation (model estimation). Evaluation of model fit was made using multiple fit indices. Goodness of fit index (GFI) and adjusted goodness of fit index (AGFI) of values >90 and root mean square error of approximation (RMSEA) of <0.06 were taken as a cut-off point (model testing)[43]. Re-specification of the hypothesized model, guided by modification indices, was made when plausible and theoretically justifiable. The total model R-square indicates the percentage of variance explained in the dependent variable (TSI).

### Ethical considerations

The ethical review board of the University of Gondar reviewed and approved the study.Permission was obtained from local administrations in the study area. Oral informed consent was sought from study subjects.The respondents were invited to participate voluntarily after informing them the potential benefits and harms involved in the study, the confidentiality and the possibility of withdrawing from the interview even without giving reasons. The University’s ethical review board approved the consent procedure. All interviews were made in private settings.

## Results

### Respondents’ characteristics

A total of 2,158 mothers were included in the study. The majority, 1,982(92%), of the respondents resided in rural areas. Nearly a third, 725 (33.6%),were young women (15–24). The median (IQR) age of the women was 27(10) years.

The median number of children ever born (IQR) among the respondents was 3 (1). More than half, 1,167 (54.1%), of the women delivered in the past six months prior to the data collection time. More than half, 656 (56.2%), of them delivered at home. The majority of the women, 1,280 (59.3%), attended two or more antenatal care during the current or last pregnancy. The majority of the women, 1,775 (82.3%), lived within a 10 kilometer radius, with an average distance (**±**SD) of 6.9 (±6.3) Kilometers, from the nearest health facility.

Farming was the main source of income for more than 93% of the households. Nearly two thirds, 1,413 (65.5%), of the women and half, 1,069(49.5%), of their partners did not go to school. Nearly all of the study participants, 2,126(98.5%), were followers of Orthodox Christianity **(Table 1).**

**Table 1:** Socio-demographic profile of study participants, northwest Ethiopia, March 2016.

Variables	No	Percent
**Place of Residence (n = 2158)**		
Urban	173	8
Rural	1985	92
**Maternal age (n = 2158)**		
15–19 years	221	10.2
20–24 years	504	23.4
25–29 years	576	26.7
30–39 years	726	33.6
40–49 years	131	6
Median (IQR)	27 (10)	
**Number of children ever born (n = 2158)**		
0	317	14.7
1	230	10.7
2	376	17.4
3	348	16.1
_4 or more	887	41.1
Median (IQR) (SD)	3(1))	
**Number of ANC visits in the current or last pregnancy (n = 2158)**		
0	576	26.7
1	302	14
2 or more	1280	59
**Marital status (n = 2158)**		
Single	34	1.6
Married	2046	94.8
Others (divorced, separated, widowed)	78	3.6
**Occupation of mother (n = 2158)**		
Farmer	2024	93.8
Merchant	50	2.4
Others (daily laborer, government employee etc)	84	3.8
**Occupation of father (n = 2158)**		
Farmer	2041	94.6
Others (Merchant daily laborer, government employee)	117	5.4
**Mothers School Attendance (2158)**		
Yes	745	34.5
No	1413	65.5
**Educational status of mother (n = 2158)**		
**Illiterate (none)**	1413	65.5
Able to read and write	68	3.2
1-4^th^ grade	235	10.9
5-8^th^ grade	279	12.9
9-10^th^ grade	141	6.5
Higher than 10^th^ grade (11-12^th^ grade and higher education)	22	1
**Fathers school attendance (n = 2158)**		
Yes	1089	50.5
No	1069	49.5
**Educational status of father (n = 2158)**		
**Illiterate (None)**	1069	49.5
Able to read and write	256	11.9
1-4^th^ grade	381	17.7
5-8^th^ grade	302	14
9-10^th^ grade	117	5.4
Higher than 10^th^ grade	33	1.6
**Religion (n = 2158)**		
Orthodox Christianity	2126	98.5
Muslim	32	1.5

The socio-economic status of each women was assigned a standardized score. All the samples were ranked according to that score which was then divided into quintiles. The first principal components for the common, urban and rural wealth indices explained 16.2%, 20.7% and 17% of the total variances in the dataset.

### Evaluation of the proposed model

#### Model fit indices

Initial model testing did not meet cutoff points for the selected fit indices; GFI, AGFI and RMSEA were 0.901, 0.879 and 0.69, respectively. Improvement in the model fit indices was shown when freeing the restriction between the error terms of selected items that measure the same construct. Shared influences other than the underlying constructs could explain the correlation between the error variances.

The revised model fitted with the sample data very well. Goodness of fit index (GFI) was 0.934 and adjusted goodness of fit index (AGFI) was 0. 915. Root Mean Error of Approximation (RMSEA) was 0.057. The commonly applied test (Chi square test) was ignored since it is sensitive to large sample size[44].

#### Measurement model

Confirmatory factory analysis was done with maximum likelihood estimation to test the significance of the hypothesized measurement model. The items used to measure the constructs were all significant at p value <0.001. This shows the significance of the items in measuring their respective underlying constructs **(Table 2).**

**Table 2:** Parameter estimates of items of latent variables used in the hypothesized measurement model, northwest Ethiopia, March 2016.

Code	Constructs and indicators	Standardized factor loadings
	**Knowledge of danger signs (KDS) (n = 2158)**	
Q404	Inability to suck is a danger sign	0.62***
Q405	Fever is a danger sign	0.62***
Q406	Low temperature is a danger sign	0.46***
Q407	Abnormal body movement is a danger sign	0.68***
Q408	Fast breathing is a danger sign	0.62***
Q409	Difficulty in breathing is a danger sign	0.70***
Q410	Red, swollen eyelids and pus discharge from the eyes are danger signs	0.61***
Q411	Redness, pus or foul odor around the cord or umbilicus are danger signs	0.60***
Q412	Yellow skin, soles and palms is a danger sign	0.53***
Q413	Floppiness or absence of movement unless stimulated is a danger sign	0.57***
	**Positive Perceived Behavior toward health care providers (PPBHCP) (n = 2158)**	
Q414	Health care providers at the health center /hospital provide confidential care	0.46***
Q415	Health care providers at health center/hospital discriminate patients (reverse coded)	0.73***
Q416	Health care providers at the health center/hospital physically abuse patients (reverse coded)	0.80***
Q417	Health care providers at the health center/hospital create unnecessary delays to give care (reverse coded)	0.57***
Q418	Health care providers at the health center/hospital give us enough time to listen to our worries	0.62***
Q419	Health care providers at the health center/hospital explain procedures to us before treatment	0.55***
	**Household level mother women’s Empowerment (HLWE) (n = 2158)**	
Q420	My husband/partner is always supportive if I make decisions to take my sick newborn to treatment	0.8***
Q421	I can make decisions concerning household resources and my family	0.82***

*** indicates p<0.001

#### Structural model

**Fig 2:**
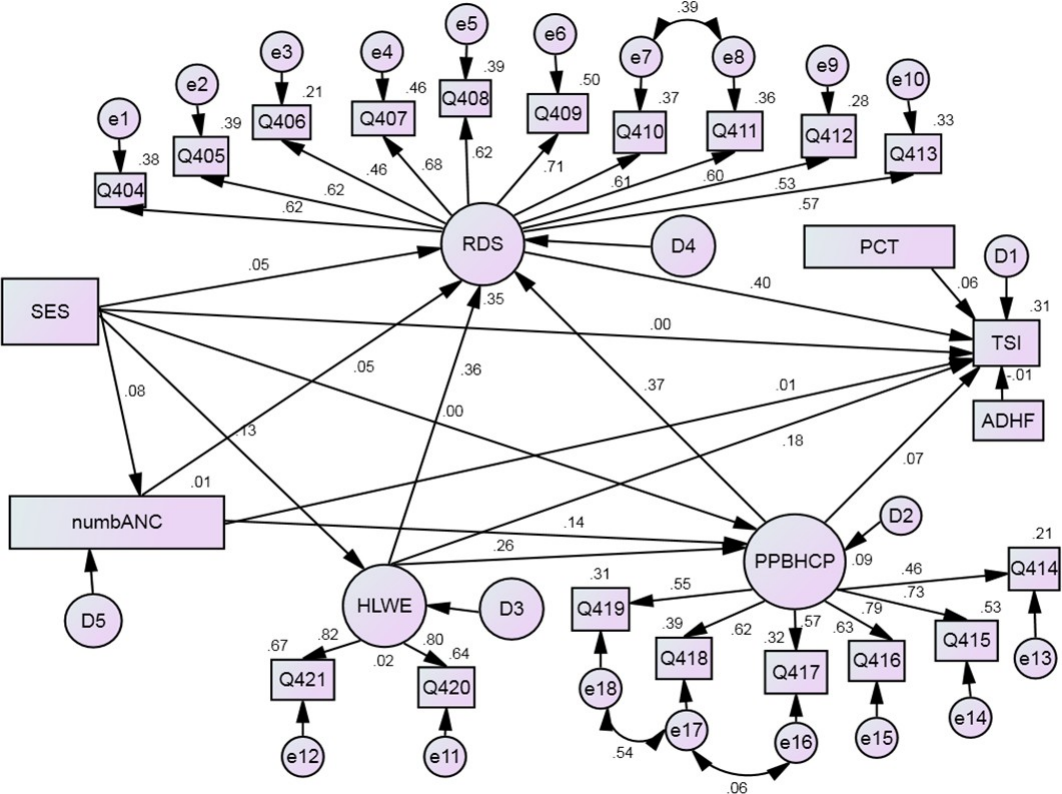
Revised model and standardized estimates for maternal treatment seeking intention for neonatal danger signs northwest Ethiopia, 2016. Path coefficient (above or right side of arrows), factor loadings (above or right side of arrows), and R^2^ (next to observed variables and on the right and above latent variables).

The standardized path coefficients show the direction and magnitude of association between two variables. Accordingly, knowledge of neonatal danger signs (KDS), positive perceived behavior of health care providers (PPBHCP), household level woman empowerment (HLWE), and perceived cost of treatment (PCT) showed direct, positive and significant association with treatment seeking intention(TSI) (β = 0.41, p<0.001, β = 0.08, p<0.002, β = 0.18, p<0.001, and β = 0.06,p<0.002, respectively). Socioeconomic status (SES) was not directly associated with TSI. However, it indirectly influenced TSI through three pathways; KDS, number of ANC attendance, and HLWE (β = 0.05, p<0.05, β = 0.08, p<0.001 and β = 0.13, p<0.001, respectively). Number of antenatal care was not directly associated with TSI. But indirectly, it affected TSI through its direct, significant and positive association with KDS and PPBHCP (β = 0.05, p<0.05, β = 0.14, p<0.001, respectively). PPBHCP and HLWE also showed indirect associations with TSI through their direct, significant and positive association with KDS (β = 0.37, p<0.001, β = 0.36, p<0.001, respectively). Average distance to health facilities (ADHF) was not associated with TSI. All in all, 31% of the variation in TSI was explained by the model (**Table 3 and Fig 2**).

**Table 3:** Standardized estimated parameters for the structural model (Equation 1–5), northwest Ethiopia, 2016.

Path (n = 2158)	Parameter	Standardized estimate
KDS—>TSI	β_1_	0.40**
PPBHCP—->TSI	β_2_	0.08**
SES—>TSI	β_3_	-0.002
Number of ANC attendance—->TSI	β_4_	0.015
HLWE—->TSI	β_5_	0.18**
ADHF—>TSI	β_6_	-0.009
PCT—>TSI	β_7_	0.057**
PPBHCP——>KDS	β_8_	0.37**
SES—->KDS	β_9_	0.05**
Number of ANC attendance—>KDS	β_10_	0.05**
HLWE—->KDS	β_11_	0.36**
SES—->PPBHCP	β_12_	0.004
Number of ANC attendance—->PPBHCP	β_13_	0.14**
HLWE—->PPBHCP	β_14_	0.26**
SES—->HLWE	β_15_	0.13**
SES—->number of ANC attendance	β_16_	0.08**

** = p<0.05, broken arrows show direction of influence

## Discussion

Several studies reported that delay in treatment seeking and not seeking any care at all significantly contribute to large number of child deaths in developing countries[45–49]. Treatment seeking is a multidimensional phenomenon influenced by many factors[50]. Hence, better understanding of the dimensions and their relationships contribute to reductions in child mortality.

This study hypothesized a theoretical model of relationships among variables that determine TSI of mothers for newborn danger signs. The model fitted the sample data and explained 31% of the variance in TSI. This suggests the applicability of the model in explaining TSI in similar settings.

According to the results of this study, PPBHCP, HLWE, KDS and PCT were directly associated with TSI. Indirectly, number of ANC attendance, PPBHCP and HLWE affected TSI through their direct effects on KDS. SES did not show a direct effect on TSI. However, it showed effect on TSI through three different pathways; KDS, number of ANC and HLWE.

The direct effect of PPBHCP on TSI is in line with a study in India which reported the influence of clients’ perceptions of health care providers behavior on service satisfaction and treatment seeking[51]. Other studies also showed significant association between how consumers view health care providers’ and treatment seeking behavior[50,52]. However, these studies assessed the direct effect of caretakers’ perception of health care providers on treatment seeking.

A systematic review of studies showed negative influence of health service providers’ behavior on provider-patient communication deterring patients from asserting their needs for information and explanations [53]. The indirect influence of PPBHCP on TSI through KDS could be due to the importance of health care providers as source of health information to mothers. If negative perception toward health care providers affects provider-clients communication, there will be reduced opportunity for health workers to inform clients about dangers signs.

Disrespect and abuse of patients by health care providers may also act as more powerful deterrents to care seeking than geographic and financial obstacles[54]. Since PPBHCP in this study is measured by the dimensions of disrespect and abuse, low scores on PPBHCP relates to low treatment seeking given its direct and indirect association with TSI. In this regard, the Health Sector Transformation Plan (HSTP) of the government of Ethiopia gives focus on compassionate and respectful care (CRC) [55]. The focus on CRC may contribute to better provider–client communication, maternal knowledge of danger signs and improved treatment seeking for newborns danger signs.

The positive and significant association between KDS and TSI is also in agreement with studies conducted in Uganda and India[51,56]. Both studies recommended, based on their findings, the importance of raising awareness on neonatal danger signs to improve treatment seeking. A study in Nigeria and Ethiopia also reported significant association between maternal knowledge of danger signs in children and subsequent treatment seeking behavior[25,57]. This calls for the need to strengthen maternal awareness of newborn danger signs using available contact points within the community and during antenatal, delivery and postnatal care.

In our study, number of ANC attendance was associated with KDS and PPBHCP. This could be attributable to the opportunities created during ANC visits to meet with health care providers. This is because ANC visits allow mothers to interact and form opinions about health care providers. ANC visits can be used as opportunities to pass messages on about newborn danger signs and create lasting positive attitude towards health care providers through promotion of compassionate and respectful care.

This study also showed a significant positive effect of HLWE on maternal TSI for newborn danger signs. Even though some studies indicate that empowered women are more likely to seek treatment compared to women who are not [34,35], the studies didn’t differentiate whether empowerment of women equally affect treatment seeking for their newborns. Some studies reported the direct effects of income and distance on treatment seeking behavior[11,33]. However, these studies reported no significant direct relationship between SES and ADHF with TSI. The difference in the case of income could be attributed to the difference in which income and SES were assessed in our study and the other studies. In addition, these studies assessed the effect of income on treatment seeking behavior (the practice), whereas our study assessed treatment seeking intention; what the mother would do if she were to see danger signs in newborns.

Although SES was not directly affecting mothers’ TSI, it was significantly associated with number of ANC attendance, KDS and HLWE influencing TSI indirectly. This suggests that better SES can serve as an enabling factor in influencing treatment seeking through its impact on other factors

### Limitation of the study

This study used a Five Point Likert Scale for the sake of simplicity. This is because higher scales (i.e. 10 or more scales) could be difficult to answer taking the differences in the scale in to account given the level of illiteracy in the study area. However, higher scales of measurement could have captured additional variances which may have resulted in higher correlation coefficient estimates. Hence, there could be a chance of committing a type II error.

Treatment seeking intention has been measured as a proxy to actual practice. Treatment seeking intention and recognition of danger signs could be different when mothers actually have newborns with danger signs. Hence, a prospective follow up study could give more information than a cross sectional study.

## Conclusion

The proposed theoretical model can be used to model treatment seeking intention in similar settings. The model indicates the network of factors and their direct and indirect pathways of influence on TSI for neonatal danger signs.

It is very important to raise awareness of danger signs at all levels including messages on neonatal danger signs during antenatal care to improve mothers’ knowledge of newborn danger signs. Implementing compassionate and respectful care and empowering women should be strengthened in Ethiopia to improve treatment seeking for newborn illnesses.
